# Mechanisms of Acquired Androgen Independence during Arsenic-Induced Malignant Transformation of Human Prostate Epithelial Cells

**DOI:** 10.1289/ehp.9630

**Published:** 2006-11-20

**Authors:** Lamia Benbrahim-Tallaa, Mukta M. Webber, Michael P. Waalkes

**Affiliations:** 1 Inorganic Carcinogenesis Section, Laboratory of Comparative Carcinogenesis, National Cancer Institute at the National Institute of Environmental Health Sciences, National Institutes of Health, Department of Health and Human Services, Research Triangle Park, North Carolina, USA; 2 Department of Medicine and; 3 Department of Zoology, Michigan State University, East Lansing, Michigan, USA

**Keywords:** androgen-independent, androgen receptor, arsenic, cancer progression, hormone refractory, malignant transformation, MAP kinase, prostate, Ras

## Abstract

**Background:**

Prostate cancer progression often occurs with overexpression of growth factors and receptors, many of which engage the Ras/mitogen-activated protein MAP kinase (MAPK) pathway.

**Objectives:**

In this study we used arsenic-transformed human prostate epithelial cells, which also show androgen-independent growth, to study the possibility that chronic activation of Ras/MAPK signaling may contribute to arsenic-induced prostate cancer progression.

**Methods:**

Control and chronic arsenic–transformed prostate epithelial cells (CAsE-PE) were compared for Ras/MAPK signaling capacities using reverse transcription–polymerase chain reaction and Western blot analyses.

**Results:**

We found activation of HER-2/neu oncogene in transformed CAsE-PE cells, providing molecular evidence of androgen independence in the transformed cells. CAsE-PE cells displayed constitutively increased expression of unmutated K-Ras (6-fold), and the downstream MAP kinases A-Raf and B-Raf (2.2-fold and 3.2-fold, respectively). There was also increased expression of phosphorylated MEK1/2 and Elk1 in the transformant cells. The MEK1/2 inhibitor, U0126, blocked PSA overexpression in CAsE-PE cells.

**Conclusion:**

Thus, arsenic-induced malignant transformation and acquired androgen independence are linked to Ras signaling activation in human prostate epithelial cells. Chronic activation of this pathway can sensitize the androgen receptor to subphysiologic levels of androgen. This may be important in arsenic carcinogenesis and provide a mechanism that may be common for prostate cancer progression driven by diverse agents.

Prostate cancer is a common malignancy and a leading cause of cancer death in the United States (Crawford 2003). The molecular mechanisms underlying its development and progression remain poorly understood. Prostate cancer usually begins as an androgen-dependent tumor that may undergo clinical regression in response to pharmacologic or surgical strategies that reduce circulating testosterone concentrations or block androgen actions (Kyprianou and Isaacs 1988). Despite this treatment, prostate tumors often reappear as androgen-independent or hormone-refractory cancers (Westin and Bergh 1998). The molecular basis for this acquired androgen independence is poorly defined.

Most androgen-independent prostate tumors overexpress androgen receptor (AR) as well as androgen-activated gene products such as prostate-specific antigen (PSA) (Kim and Coetzee 2004). In fact, hormone-refractory prostate cancer cells often can maintain functional AR signaling despite greatly reduced levels of circulating testosterone as, for instance, with orchiectomy (Deutsch et al. 2004) because of AR overexpression. High levels of AR can correlate with prostatic cancer progression in many cases (Lee and Tenniswood 2004), and AR gene amplification is observed in 20–30% of all hormone-refractory prostate cancers (Linja et al. 2001). A role for AR in prostate cancer progression is supported by the correlation between its overexpression and the expression of androgen-regulated genes (Gregory et al. 1998). Up-regulation of AR clearly increases sensitivity to low androgen levels, leading to ligand-dependent downstream androgen-regulated gene expression and prostate tumor recurrence. In fact, the term “androgen-independent” prostate cancer is somewhat of a misnomer, because it often involves androgen hypersensitivity rather than true independence (Gregory et al. 2001).

There are also mechanisms for AR activation in recurrent or advanced prostate cancers in which overexpression is not obligatory. These include altered growth factor–induced phosphorylation (Craft et al. 1999; Sadar et al. 1999) and AR mutations that broaden ligand specificity (Newmark et al. 1992; Tan et al. 1997). Indeed, some hormone-refractory prostate cancers appear to recruit non-steroidal signal transduction pathways that activate AR even in the face of severe androgen deprivation (Taplin and Balk 2004). Thus, a “by-pass” of the requirement for androgen to interact with AR for activation of downstream events can occur in prostate cancer with acquired androgen independence (Feldman and Feldman 2001; Gleave et al. 1999). Indeed, HER-2/neu, a member of the EGF-receptor family of receptor tyrosine kinase, can activate AR-dependent genes in the absence of AR-ligand interactions (Culig et al. 1994; Yeh et al. 1999). In fact, HER-2/neu, when overexpressed, not only activates the AR pathway in the absence of ligand but also synergizes with low levels of androgen to “superactivate” this pathway (Craft et al. 1999). HER-2/neu overexpression often occurs in advanced prostate cancers (Ady et al. 2004), and stimulates growth and PSA overproduction (Craft et al. 1999). Thus, increased expression of HER-2/neu in prostate cancer cells allows androgen-independent growth (Craft et al. 1999; Culig and Bartsch 2006).

Inorganic arsenic, a common environmental contaminant and human carcinogen [International Agency for Research on Cancer (IARC) 2004; National Research Council (NRC) 1999; National Toxicology Program (NTP) 2000], has been associated with human prostate cancer (Chen and Wang 1990; Lewis et al. 1999; NRC 1999; Wu et al. 1989). In addition, we have shown that arsenic can precipitate events leading to malignant transformation of human prostate epithelial cells *in vitro* (Achanzar et al. 2002) and can affect prostate cancer cell progression (Benbrahim-Tallaa et al. 2005b). In this regard, the immortalized, nontumorigenic human prostate epithelial cell line (RWPE-1) is malignantly transformed by chronic low-level arsenic to produce CAsE-PE (chronic arsenic–exposed prostate epithelial) cells which form tumors resembling aggressive prostate carcinoma on injection into nude mice (Achanzar et al. 2002) and show characteristics of advanced, androgen-independent prostate cancer (Benbrahim-Tallaa et al. 2005b). Arsenic-induced androgen independence in CAsE-PE cells is not associated with AR overexpression or any apparent loss of ligand specificity (Benbrahim-Tallaa et al. 2005b), indicating that arsenic impacts progression through other mechanisms. Importantly, wild-type K-Ras activation was strongly correlated with arsenic-induced transformation in CAsE-PE cells (Benbrahim-Tallaa et al. 2005a). In this regard, Ras is a component of virtually all the signaling pathways shown to be up-regulated in advanced prostate cancer (Hoa et al. 2002; Rizos et al. 1999; Yu et al. 1993). Although Ras is infrequently mutated in prostate cancer, overexpression of wild-type Ras can be sufficient to induce malignant transformation in human prostate epithelial cells (Weber and Gioeli 2004).

Thus, the present study investigated in detail the molecular mechanism by which arsenic impacts prostate tumor cell progression using CAsE-PE cells. The results indicate that overexpression of HER-2/neu and K-Ras, resultant mitogen-activated protein kinase (MAPK) pathway activation and downstream Raf activation are prominent features. Thus, it appears arsenic-induced malignant transformation precipitates up-regulation of Ras, which in turn allows a by-pass of AR to induce androgen independence in human prostate epithelial cells.

## Material and Methods

### Chemicals and reagents

We purchased sodium arsenite (NaAsO_2_ ) from Sigma Chemical Co. (St. Louis, MO), and keratinocyte serum-free medium (K-SFM), epidermal growth factor (EGF), bovine pituitary extract (BPE), 100 X antibiotic-antimycotic mixture, and TRIzol Reagent from Life Technologies, Inc. (Grand Island, NY). The mouse monoclonal anti-K-Ras and the mouse monoclonal anti-actin were purchased from Oncogene Research Products (Cambridge, MA). We purchased the rabbit polyclonal anti-HER-2/Erb2, anti-A-Raf, anti-B-Raf, anti-MEK1/2, anti-Elk1, and horseradish per-oxidase-conjugated secondary antibody from Cell Signaling Technology (Beverly, MA), and the Bradford Protein Assay from Bio-Rad Laboratories (Hercules, CA).

### Cells and cell culture

Control (untransformed) RWPE-1 cells were originally derived from normal human prostate epithelial cells and are immortalized but nontumorigenic (Bello et al. 1997; Webber et al. 1997). Unless otherwise noted, cells were grown in K-SFM containing 50 μg/mL BPE and 5 ng/mL EGF, supplemented with antibiotic/antimycotic mixture. K-SFM containing BPE and EGF is henceforth termed “complete medium.” The steroid-reduced media is defined as a medium without complement. The BPE is likely the major source of steroids in complete medium. Cultures were incubated at 37°C in a humidified atmosphere containing 5% carbon dioxide and passaged weekly. Cells were exposed continuously to 5 μM arsenite (as sodium arsenite). The arsenic-exposed cells were designated as CAsE-PE cells to distinguish them from the parental control cells. Parallel cultures grown in arsenic-free medium provided passage-matched controls. At 29 weeks of exposure, CAsE-PE cells produce malignant tumors after inoculation into nude mice (Achanzar et al. 2002). We previously showed that a clear transition from the androgen-sensitive to androgen-independent state occurs during arsenic-induced malignant transformation of human prostate epithelial cells (Benbrahim-Tallaa et al. 2005b).

### RNA extraction and RT-PCR

We isolated total RNA using TRIzol reagent by manufacturer’s instructions. Reverse transcription–polymerase chain reaction (RT–PCR) was performed using a TITANIUM one-step kit (Clontech, San Jose, CA) and a GeneAmp PCR system 9700 (Applied Biosystems, Foster City, CA) according to the kit’s instructions. Amplification conditions were as follows: 60 min at 50°C and 5 min at 94°C followed by 35 cycles each for 1 min at 94°C, 1 min at 54°C, 1 min at 72°C. We used 1 μg of total RNA in each amplification. Primers were designed for PSA and β -actin and were synthesized by Invitrogen as follows: PSA (5′-GAGGTCCACACACTGAAGTT-3′ and 5′-CCTCCTGAAGAATCGATTCCT-3′), product size: 214 bp; β -actin (5′-AGA-GATGGCCACGGCTGCTT-3′ and 5′-ATTTGCGGTGGACGATGGAG-3′), product size: 460 bp. PCR products were electrophoresed on 1.7% agarose gels and the gel image captured and quantified with a Gel Doc 2000 System equipped with TDS Quantity One software (Bio-Rad Laboratories). The level of β -actin was used to normalize results.

### Western blot analysis

We isolated total proteins using M-PER reagent (Pierce, Rockford, IL) as directed by the manufacturer. We determined protein concentration using the Bradford assay, and 20–40 μg of each sample were electrophoresed and transferred to nitrocellulose membranes (Invitrogen). Immunoblotting was performed using the human K-Ras antibody at a 1:1,000 dilution; horseradish peroxidase–conjugated anti-mouse secondary antibody at a 1:5,000 dilution; HER-2/neu antibody at 1:1,000 dilution; A-Raf antibody at 1:1,000 dilution; B-Raf antibody at 1:1,000 dilution; Phospho-MEK1/2 antibody at 1:1,000 dilution; and phospho-Elk1 antibody at 1:1,000 dilution; horseradish peroxidase–conjugated anti-rabbit secondary antibody at a 1:5,000 dilution; and SuperSignal West Pico Chemiluminescent Substrate (Pierce). Signals were visualized by exposure to Hyperfilm (Amersham). Densitometric analysis was performed using Quantity One software (Bio-Rad).

### Statistical analysis

All data are represented as mean ± SE derived from three or more independent experiments. Statistical significance was determined by the Student *t*-test or ANOVA followed by Dunnett’s *t*-test as appropriate, with *p* ≤ 0.05 considered statistically significant.

## Results

### AR activity and malignant transformation leading to acquired androgen independence

**Figure 1:**
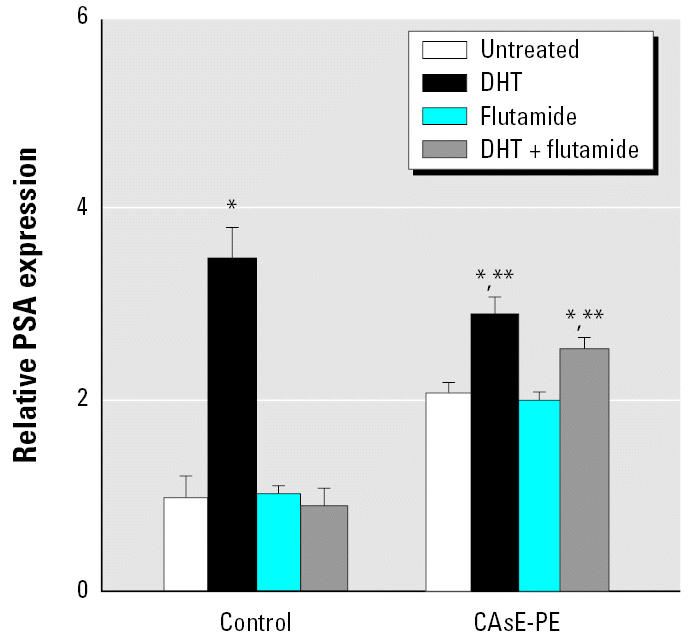
The effect of flutamide on the PSA mRNA expression of control RWPE-1 and CAsE-PE cells. Control and CAsE-PE were grown in complete medium. After 48 hr in steroid-reduced medium defined as K-SFM without BPE and EGF, cells were fed with fresh steroid-reduced medium with or without 5 μg/mL flutamide in the presence or absence of 0.1 μM DHT. RNA was isolated and subjected to RT-PCR analysis using a set of primers designed to amplify PSA and β -actin gene products. PCR products were separated on a 1.7% agarose gel. The data shown are expressed as means (*n* = 3); error bars represent SE. *Significantly different from untreated, cell-line–matched cells. **Significantly different from control cells treated with DHT or DHT plus flutamide.

Arsenic precipitates events leading to rapid growth and greatly reduced androgen dependence during malignant transformation of human prostate epithelial cells *in vitro* (Benbrahim-Tallaa et al. 2005a, 2005b). AR expression or ligand specificity played a minimal role in this arsenic-induced prostate cancer cell progression (Benbrahim-Tallaa et al. 2005b). In fact, AR was expressed at essentially the same level in both control and CAsE-PE cells (Benbrahim-Tallaa et al. 2005b). To further assess the activity of AR in these cells, we examined androgen-induced gene expression through AR stimulation. In this case we examined PSA expression, which is activated by androgens through AR. As is typical with prostate malignancies, CAsE-PE cells expressed significantly more PSA (2.1-fold, *p* < 0.05) than control cells (Figure 1). However, compared with respective untreated cells, a marked 3.5-fold increase in cellular PSA mRNA levels occurred with dihydrotestosterone (DHT) treatment in control RWPE-1 cells, whereas levels increased only 0.8-fold in CAsE-PE cells. Indeed, DHT-induced increases in PSA were to a significantly lower maximal level in CAsE-PE cells compared with control cells (Figure 1). Thus, AR overexpression was clearly not required for the steroid-independent growth in CAsE-PE cells. Because androgen-independent prostate cancers often become resistant to antiandrogens, we tested the effect of the antiandrogen flutamide on DHT-stimulated PSA mRNA levels in control and CAsE-PE cells. A DHT-stimulated PSA mRNA level was completely suppressed by flutamide in control cells (Figure 1). On the other hand, in CAsE-PE cells the androgen-stimulated PSA secretion was blocked only partially by flutamide. Collectively, CAsE-PE cells responded differently to DHT and flutamide, both of which are thought to act through direct interaction with AR.

### HER-2/neu activation with arsenic-induced acquired androgen independence

**Figure 2:**
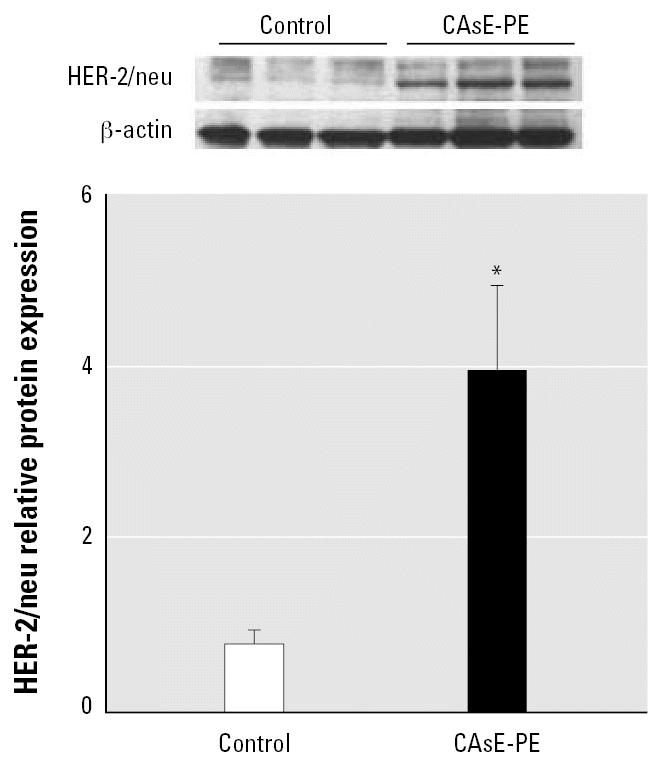
Analysis of the effect of chronic arsenic exposure of prostate epithelial cells on HER-2/neu protein expression. CAsE-PE cells were first derived by exposing RWPE-1 cells to 5 μM sodium arsenite for 30 weeks. Proteins were isolated from control and arsenic-transformed CAsE-PE cells and subjected to Western blot analysis. Upper panel is a representative blot, whereas the lower panel is densitometric analysis normalized to β -actin. Densitometric data are given as fold-control and expressed as means (*n* = 3); error bars represent SE. *Significantly different from control.

Several reports have described the involvement of HER-2/neu tyrosine kinase—a type I growth factor receptor tyrosine kinase—in activating the AR through the MAPK pathway (Ady et al. 2004; Craft et al. 1999), and HER-2/neu expression has been implicated in the progression of prostate cancer to a hormone independence (Yeh et al. 1999; Wen et al. 2000). Therefore, the level of HER-2/neu expression in control (RWPE-1) cells was compared with androgen-independent, arsenic-transformed CAsE-PE cells (Figure 2). HER-2/neu protein in transformed CAsE-PE cells was expressed at levels > 400% greater than control. This is consistent with the aggressive nature and androgen independence of CAsE-PE cells.

### Arsenite enhances K-Ras gene expression during malignant transformation

**Figure 3:**
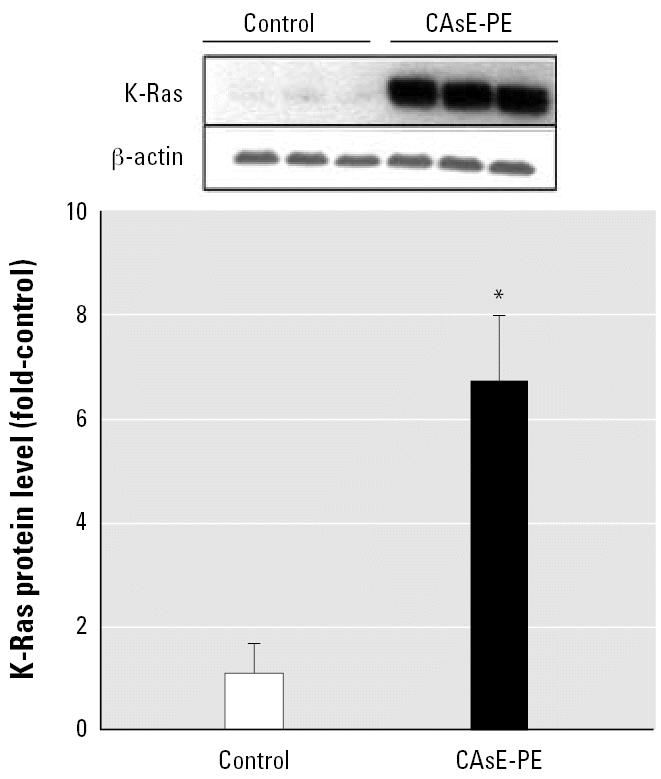
Analysis of the effect of chronic arsenic exposure of prostate epithelial cells on K-ras protein expression. CAsE-PE cells were first derived by exposing RWPE-1 cells to 5 μM sodium arsenite for 30 weeks. Proteins were isolated from control and arsenic-transformed CAsE-PE cells and subjected to Western blot analysis. Upper panel is a representative blot, whereas the lower panel is densitometric analysis normalized to β -actin. Densitometric data are given as fold-control and expressed as means (*n* = 3); error bars represent SE. *Significantly different from control.

Because Ras-related pathways are often related to androgen independence in prostate cancer, this potential oncogene was studied in detail. K-Ras protein level in CAsE-PE cells was greatly increased (6-fold) compared with control (Figure 3). We previously showed that K-Ras overexpression precedes and remains elevated with malignant transformation of CAsE-PE cells (Benbrahim-Tallaa et al. 2005a).

### MAPK pathway correlates with prostate cancer cell progression

**Figure 4:**
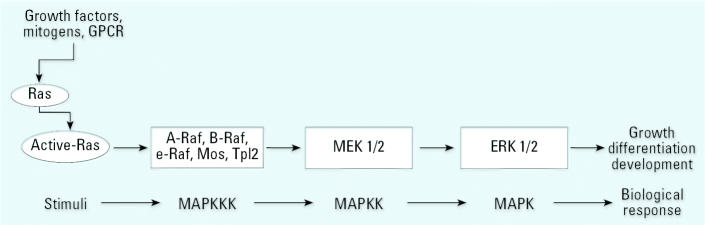
MAPK pathway. Abbreviations: GPCR, G protein coupled receptors; GTPase, guanosine triphosphatases. MAPK are a family of serine–threonine protein kinases widely conserved among eukaryotes and are involved in many cellular programs such as cell proliferation, cell differentiation, cell movement, and cell death. MAPK signaling cascades are organized hierarchically into three-tiered modules. MAPKs (Erk) are phosphorylated and activated by MAPK-kinases (MAPKKs), (MEK1/2), which in turn are phosphorylated and activated by MAPKK-kinases (MAPKKKs), (Raf). The MAPKKKs are in turn activated by interaction with the family of small GTPases and/or other protein kinases (Ras, Rap1), connecting the MAPK module to cell surface receptors or external stimuli. An activated Erk dimer can regulate targets in the cytosol and also translocate to the nucleus where it phosphorylates a variety of transcription factors regulating gene expression.

**Figure 5:**
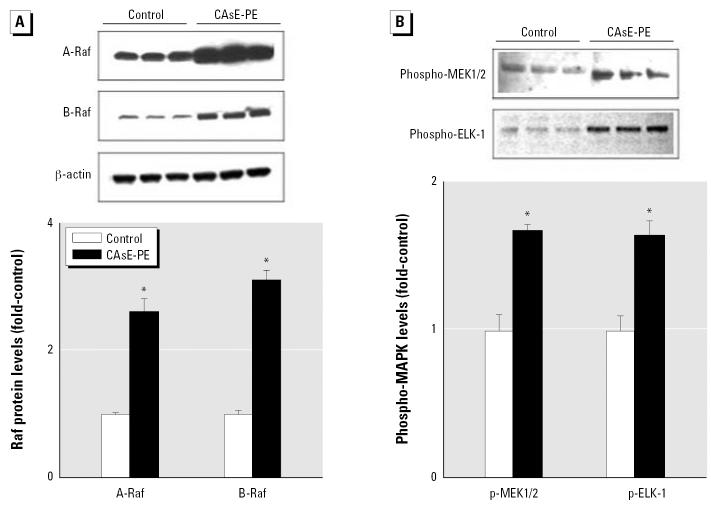
Analysis of the effect of chronic arsenic exposure of prostate epithelial cells on MAPK activation. ELK, E-26-like protein-1. CAsE-PE cells were first derived by exposing RWPE-1 cells to 5 μM sodium arsenite for 30 weeks. Proteins were isolated from control and arsenic-transformed CAsE-PE cells and subjected to Western blot analysis. (*A*) A- and B-Raf protein expression. (*B*) MEK1/2 and ELK-1 MAPK activation. Upper panel is a representative blot, whereas the lower panel is densitometric analysis normalized to β -actin. Densitometric data are given as fold-control and expressed as means (*n* = 3); error bars represent SE. *Significantly different from control.

**Figure 6:**
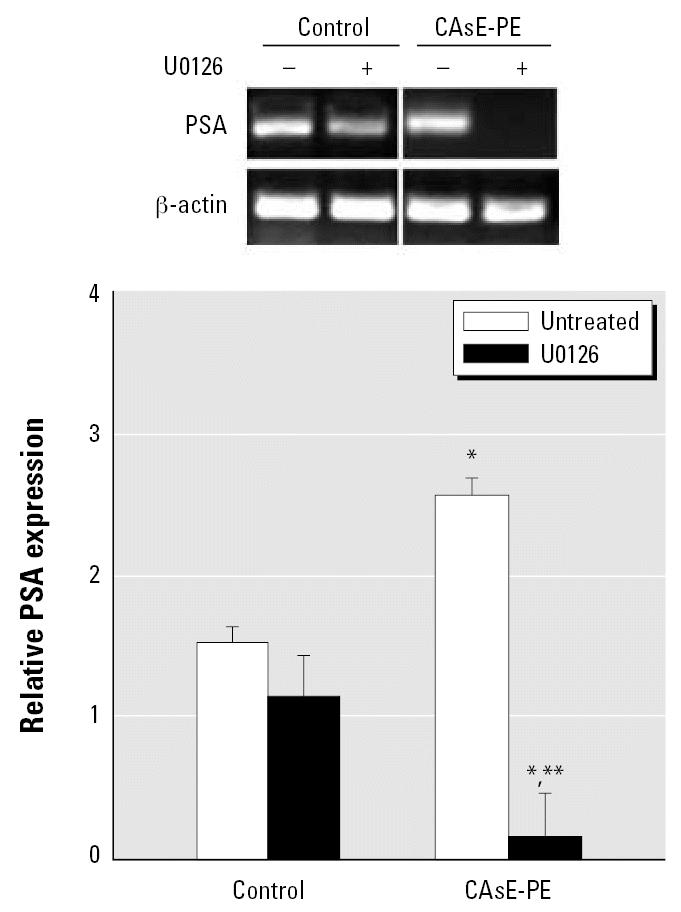
The effect of U0126, a MEK inhibitor, on PSA expression in control RWPE-1 and CAsE-PE cells. Control and CAsE-PE were grown in complete medium in the presence or absence of 20 μM U0126. RNA was isolated and subjected to RT-PCR analysis using a set of primers designed to amplify PSA and β -actin gene products. PCR products were separated on a 1.7% agarose gel. Results are normalized to β -actin. The data shown are expressed as means (*n* = 3); error bars represent SE. *Significantly different from untreated, cell-line–matched cells. **Significantly different from control cells treated with U0126.

Ras is a critical signaling molecule that controls several signaling pathways in prostate cancer. One of the best-characterized effector pathways trigged by Ras activation is the MAPK pathway (Figure 4). Thus, events downstream of Ras in CAsE-PE cells were compared with control RWPE-1. Clearly, proteins downstream of K-Ras, including A-Raf and B-Raf (both serine–threonine MAP kinases) showed greatly increased expression in CAsE-PE cells compared with control (2.6- and 3.1-fold, respectively) (Figure 5A). There was also an increased expression of phosphorylated MEK1/2 and ELK in CAsE-PE cells compared with control (Figure 5B). Thus, there is a correlation between elevated levels of active phospho-MAPK and arsenic-induced prostate cell transformation. Also, treatment with the specific MEK inhibitor, U0126, inhibited PSA expression up to 80% in CAsE-PE cells (Figure 6).

## Discussion

The normal development, growth, and survival of the prostate epithelium is regulated both by androgen and by the paracrine production of growth factors by the prostatic stroma (Feldman and Feldman 2001). However, regulatory interactions between androgens and growth factors often become distorted in prostate cancer. Early-stage prostate cancer typically requires androgen for growth and thus responds to androgen ablation (Feldman and Feldman 2001). However, following such therapy, the disease often progresses to an androgen-independent state, rendering androgen ablation ineffective (Arnold and Isaacs 2002).

Progression of prostate cancer to the frequently fatal androgen-independent disease is often associated with the elevation and autocrine production of multiple polypeptide growth factors (Gioeli et al. 1999). For example, EGF, transforming growth factor α, insulin-like growth factor 1, interleukin 6, keratinocyte growth factor, and other fibroblast growth factor family members are expressed in advanced prostate cancers and are believed to be important in fueling androgen-independent growth (Culig et al. 1994; Yeh et al. 1999). Among compounds that activate AR function in the absence of ligand or in a synergistic manner with low androgen levels, it appears the EGF receptor-related molecule, HER-2/neu, plays a critical role (Craft et al. 1999). A type I growth factor receptor tyrosine kinase, HER-2/neu is over-expressed in most epithelial malignancies (Carles et al. 2004; Ren et al. 2005; Wu et al. 2004). HER-2/neu overexpression results in enhanced growth of prostate cancer and up-regulation of PSA (Craft et al. 1999; Shi et al. 2006; Veeramani et al. 2005). Our results showed that CAsE-PE cells greatly overexpress HER-2/neu compared with control in association with malignant transformation and acquired androgen independence. HER-2/neu production is positively regulated by androgens in androgen-dependent LNCaP prostate cancer cells (Zhau et al. 1992). A transmembrane tyrosine kinase, HER-2/neu, triggers intracellular signaling cascades such as MAP and Akt kinase pathways (Culig 2004; Wen et al. 2000). Importantly, HER-2/neu promotes phosphorylation of AR at multiple sites, which results in a highly active transcriptional unit even in the presence of low androgen levels (Culig 2004; Sugita et al. 2004). Thus, it appears HER-2/neu activation is a key factor in arsenic-induced acquired androgen independence in malignant human prostate epithelial cells.

Because Ras/MAPK signaling can reduce the androgen requirement of prostate cancer cells (Bakin et al. 2003b), one would predict that stimulation of this signaling pathway might allow androgen-regulated gene expression even at very low levels of androgen. Previous studies showed that flutamide inhibits the androgen-signaling pathway in androgen-dependent but not refractory tumors (Ilagan et al. 2005). We measured endogenous PSA expression as an indicator of androgen-regulated gene expression. The antiandrogen flutamide completely abolished PSA expression in control cells but only partially attenuated PSA production in transformed CAsE-PE cells. This finding is consistent with an androgen refractory status.

Overexpression of K-Ras and activation of MAPK correlate with progression in CAsE-PE cells (Benbrahim-Tallaa et al. 2005a, 2005b). Similarly, expression of activated Ras makes LNCaP epithelial prostate cancer cell line less dependent on androgens (Bakin et al. 2003b). Also, expression of dominant negative Ras restores androgen dependence to C4-2 cells (Bakin et al. 2003a). C4-2 is a hormone-refractory derivative of the LNCaP cell line (Wu et al. 1994). Our work *in vitro* shows that the common environmental contaminant arsenic can induce malignant transformation associated with androgen independence in human prostate epithelial cells (Achanzar et al. 2002; Benbrahim-Tallaa et al. 2005b).The increase in MAPK activation in androgen-independent CAsE-PE cells in the present study is consistent with the hypothesis that prostate cancer progression induced by arsenic is associated with chronic stimulation of the Ras signaling pathway.

Treatment of cells with the MAPK inhibitor U0126 totally abolished PSA expression in CAsE-PE cells but only partially in control cells. PSA expression is normally controlled by androgen through AR. Because androgen-refractory CAsE-PE cells greatly overexpress PSA, these results clearly show MAPK activity is critical to arsenic-induced acquired androgen independence. This fortifies the concept that events downstream of AR are of critical importance to arsenic-induced prostate cancer progression.

In summary, this study indicates that arsenic-induced malignant transformation and acquired androgen independence in human prostate epithelial cells is associated with an apparent “by-pass” of the androgen requirement for AR pathway activation. The most likely basis of this androgen independence is overexpression of HER-2/neu. Activation of the Ras/MAPK pathway in CAsE-PE cells correlates with this progression to androgen independence. Indeed, blockage of MAPK inhibited androgen-regulated gene expression, highlighting the role of Ras-directed signaling pathways in arsenic-induced androgen independence. These observations indicate that this important and common environmental contaminant can potentially stimulate both initiation and progression of human prostate cancer. Progression to androgen independence is a key factor in the eventual mortality from this all too common malignancy.

## References

[b1-ehp0115-000243] Achanzar WE, Brambila EM, Diwan BA, Webber MM, Waalkes MP (2002). Inorganic arsenite induced malignant transformation of human prostate epithelial cells. J Natl Cancer Inst.

[b2-ehp0115-000243] Ady N, Morat L, Fizazi K, Soria J-C, Mathieu M-C, Prapotnich D (2004). Detection of HER 2/neu positive circulating epithelial cells in prostate cancer patients. Br J Cancer.

[b3-ehp0115-000243] Arnold JT, Isaacs JT (2002). Mechanisms involved in the progression of androgen independent prostate cancers: it is not only the cancer cell’s fault. Endocr Relat Cancer.

[b4-ehp0115-000243] Bakin RE, Gioeli D, Bissonette EA, Weber MJ (2003a). Attenuation of Ras signaling restores androgen sensitivity to hormone-refractory C4-2 prostate cancer cells. Cancer Res.

[b5-ehp0115-000243] Bakin RE, Gioeli D, Sikes RA, Bissonette EA, Weber MJ (2003b). Constitutive activation of the Ras/mitogen-activated protein kinase signaling pathway promotes androgen hypersensitivity in LNCaP prostate cancer cells. Cancer Res.

[b6-ehp0115-000243] Bello D, Webber MM, Kleinman HK, Wartinger DD, Rhim JS (1997). Androgen responsive adult human prostatic epithelial cell lines immortalized by human papillomavirus 18. Carcinogenesis.

[b7-ehp0115-000243] Benbrahim-Tallaa L, Waterland RA, Styblo M, Achanzar WE, Webber MM, Waalkes MP (2005a). Molecular events associated with arsenic-induced malignant transformation of human prostatic epithelial cells: aberrant genomic DNA methylation and K-ras oncogene activation. Toxicol Appl Pharmacol.

[b8-ehp0115-000243] Benbrahim-Tallaa L, Webber MM, Waalkes MP (2005b). Acquisition of androgen independence by human prostate epithelial cells during arsenic-induced malignant transformation. Environ Health Perspect.

[b9-ehp0115-000243] Carles J, Lloreta J, Salido M, Font A, Suarez M, Baena V (2004). Her-2/neu expression in prostate cancer: a dynamic process?. Clin Cancer Res.

[b10-ehp0115-000243] Chen CJ, Wang CJ (1990). Ecological correlation between arsenic levels in well water and age adjusted mortality from malignant neoplasms. Cancer Res.

[b11-ehp0115-000243] Craft N, Shostak Y, Carey M, Sawyers CL (1999). A mechanism for hormone-independent prostate cancer through modulation of androgen receptor signaling by the HER-2/neu tyrosine kinase. Nat Med.

[b12-ehp0115-000243] Crawford ED (2003). Epidemiology of prostate cancer. Urology.

[b13-ehp0115-000243] Culig Z (2004). Androgen receptor cross-talk with cell signaling pathways. Growth Factors.

[b14-ehp0115-000243] Culig Z, Bartsch G (2006). Androgen axis in prostate cancer. J Cell Biochem.

[b15-ehp0115-000243] Culig Z, Hobisch A, Cronauer MV, Radmayr C, Trapman J, Hittmair A (1994). Androgen receptor activation in prostatic tumor cell lines by insulin-like growth factor-I, keratinocyte growth factor, and epidermal growth factor. Cancer Res.

[b16-ehp0115-000243] Deutsch E, Maggiorella L, Eschwege P, Bourhis J, Soria JC, Abdulkarim B (2004). Environmental, genetic, and molecular features of prostate cancer. Lancet Oncol.

[b17-ehp0115-000243] Feldman BJ, Feldman D (2001). The development of androgen-independent prostate cancer. Nat Rev Cancer.

[b18-ehp0115-000243] Gioeli D, Mandell JW, Petroni GR, Frierson HF, Weber MJ (1999). Activation of mitogen-activated protein kinase associated with prostate cancer progression. Cancer Res.

[b19-ehp0115-000243] Gleave M, Tolcher A, Miyake H, Nelson C, Brown B, Beraldi E (1999). Progression to androgen independence is delayed by adjuvant treatment with antisense Bcl-2 oligodeoxynucleotides after castration in the LNCaP prostate tumor model. Clin Cancer Res.

[b20-ehp0115-000243] Gregory CW, Hamil KG, Kim D, Hall SH, Pretlow TG, Mohler JL (1998). Androgen receptor expression in androgen-independent prostate cancer is associated with increased expression of androgen-regulated genes. Cancer Res.

[b21-ehp0115-000243] Gregory CW, Johnson RT, Mohler JL, French FS, Wilson EM (2001). Androgen receptor stabilization in recurrent prostate cancer is associated with hypersensitivity to low androgen. Cancer Res.

[b22-ehp0115-000243] Hoa M, Davis SL, Ames SJ, Spanjaard RA (2002). Amplification of wild-type K-ras promotes growth of head and neck squamous cell carcinoma. Cancer Res.

[b23-ehp0115-000243] IARC (International Agency for Research on Cancer) (2004). Some Drinking Water Disinfectants and Contaminants, Including Arsenic. IARC Monogr Eval Carcinog Risk Hum.

[b24-ehp0115-000243] Ilagan R, Zhang LJ, Pottratz J, Le K, Salas S, Iyer M (2005). Imaging androgen receptor function during flutamide treatment in the LAPC9 xenograft model. Mol Cancer Ther.

[b25-ehp0115-000243] Kim J, Coetzee GA (2004). Prostate specific antigen gene regulation by androgen receptor. J Cell Biochem.

[b26-ehp0115-000243] Kyprianou N, Isaacs JT (1988). Activation of programmed cell death in the rat ventral prostate. Endocrinology.

[b27-ehp0115-000243] Lee EC, Tenniswood MP (2004). Emergence of metastatic hormone-refractory disease in prostate cancer after anti-androgen therapy. J Cell Biochem.

[b28-ehp0115-000243] Lewis DR, Southwick JW, Ouellet-Hellstrom R, Rench J, Calderon RL (1999). Drinking water arsenic in Utah: a cohort mortality study. Environ Health Perspect.

[b29-ehp0115-000243] Linja MJ, Savinainen KJ, Saramaki OR, Tammela TL, Vessella RL, Visakorpi T (2001). Amplification and overexpression of androgen receptor gene in hormone-refractory prostate cancer. Cancer Res.

[b30-ehp0115-000243] Newmark JR, Hardy DO, Tonb DC, Carter BS, Epstein JL, Isaacs WB (1992). Androgen receptor gene mutations in human prostate cancer. Proc Natl Acad Sci USA.

[b31-ehp0115-000243] NRC (National Research Council) 1999. Arsenic in Drinking Water. Washington, DC:National Academy Press.

[b32-ehp0115-000243] NTP 2000. Reports on Carcinogens. First and Subsequent 2nd–9th (1980–2000). Research Triangle Park, NC:National Toxicology Program.

[b33-ehp0115-000243] Ren SH, Wang JW, Zhang L (2005). Effects of Her-2/neu siRNA-mediated gene silencing on cell cycle and apoptosis of lung adenocarcinoma cells. Zhonghua Yi Xue Za Zhi.

[b34-ehp0115-000243] Rizos E, Sourvinos G, Arvanitis DA, Velegrakis G, Spandidos DA (1999). Low incidence of H-, K- and N-*ras* oncogene mutations in cytological specimens of laryngeal tumours. Oral Oncol.

[b35-ehp0115-000243] Sadar MD, Hussain M, Bruchovsky N (1999). Prostate cancer: molecular biology of early progression to androgen independence. Endocr Relat Cancer.

[b36-ehp0115-000243] Shi Y, Chatterjee SJ, Brands FH, Shi SR, Pootrakul L, Taylor CR (2006). Role of coordinated molecular alterations in the development of androgen-independent prostate cancer: an *in vitro* model that corroborates clinical observations. BJU Int.

[b37-ehp0115-000243] Sugita S, Kawashima H, Tanaka T, Kurisu T, Sugimura K, Nakatani T (2004). Effect of type I growth factor receptor tyrosine kinase inhibitors on phosphorylation and trans-activation activity of the androgen receptor in prostate cancer cells: Ligand-independent activation of the N-terminal domain of the androgen receptor. Oncol Rep.

[b38-ehp0115-000243] Tan J-A, Sharief Y, Hamil KG, Gregory CW, Zang D-Y, Sar M (1997). Dehydroepiandrosterone activates mutant androgen receptors expressed in the androgen-dependent human prostate cancer xenograft CWR22 and LNCaP cells. Mol Endocrinol.

[b39-ehp0115-000243] Taplin ME, Balk SP (2004). Androgen receptor: a key molecule in the progression of prostate cancer to hormone independence. J Cell Biochem.

[b40-ehp0115-000243] Veeramani S, Yuan TC, Chen SJ, Lin FF, Petersen JE, Shaheduzzaman S (2005). Cellular prostatic acid phosphatase: a protein tyrosine phosphatase involved in androgen-independent proliferation of prostate cancer. Endocr Relat Cancer.

[b41-ehp0115-000243] Webber MM, Bello D, Kleinman HK, Hoffman MP (1997). Acinar differentiation by nonmalignant immortalized human prostatic epithelial cells and its loss by malignant cells. Carcinogenesis.

[b42-ehp0115-000243] Weber MJ, Gioeli D (2004). Ras signaling in prostate cancer progression. J Cell Biochem.

[b43-ehp0115-000243] Wen Y, Hu MC, Makino K, Spohn B, Bartholomeusz G, Yan DH (2000). HER-2/neu promotes androgen-independent survival and growth of prostate cancer cells through the Akt pathway. Cancer Res.

[b44-ehp0115-000243] Westin P, Bergh A (1998). Apoptosis and other mechanisms in androgen ablation treatment and androgen independent progression of prostate cancer. Cancer Detect Prev.

[b45-ehp0115-000243] Wu HC, Hsieh JT, Gleave ME, Brown NM, Patha S, Chung LW (1994). Derivation of androgen-independent human LNCaP prostatic cancer cell sublines: role of bone stromal cells. Int J Cancer.

[b46-ehp0115-000243] Wu MM, Kuo TL, Hwang YH, Chen CJ (1989). Dose-response relation between arsenic concentration in well water and mortality from cancers and vascular diseases. Am J Epidemiol.

[b47-ehp0115-000243] Wu Y, Soslow RA, Marshall DS, Leitao M, Chen B (2004). Her-2/neu expression and amplification in early stage ovarian surface epithelial neoplasms. Gynecol Oncol.

[b48-ehp0115-000243] Yeh S, Lin HK, Kang HY, Thin TH, Lin MF, Chang C (1999). From HER2/neu signal cascade to androgen receptor and its coactivators: a novel pathway by induction of androgen target genes through MAP kinase in prostate cancer cells. Proc Natl Acad Sci USA.

[b49-ehp0115-000243] Yu M, Leav BA, Leav I, Merk FB, Wolfe HJ, Ho SM (1993). Early alterations in ras protooncogene mRNA expression in testosterone and estradiol-17 beta induced prostatic dysplasia of Noble rats. Lab Invest.

[b50-ehp0115-000243] Zhau HE, Wan DS, Zhou J, Miller GJ, von Eschenbach AC (1992). Expression of c-erb B-2/neu proto-oncogene in human prostatic cancer tissues and cell lines. Mol Carcinog.

